# The MEOPAeDent trial protocol—an observational study of the Equimolar Mixture of Oxygen and Nitrous Oxide (EMONO) effects in paediatric dentistry

**DOI:** 10.1186/s12903-019-0732-6

**Published:** 2019-03-07

**Authors:** Tony Prud’homme, Sylvie Dajean-Trutaud, Morgane Rousselet, Fanny Feuillet, Marjorie Carpentier-Cheraud, Olivier Bonnot, Isabelle Hyon, Marie Grall-Bronnec, Serena Lopez-Cazaux, Caroline Victorri-Vigneau

**Affiliations:** 1 Faculty of Dental Surgery, Pediatric Dentistry Department, CHU Nantes, 1 place Alexis Ricordeau, BP 84215, 44042 Nantes, Cedex 1, France; 2 Université Nantes, Nantes, France; 3 Addictology and Psychiatry Department, CHU Nantes, Nantes, France; 4 Pharmacology Department – Addictovigilance, CHU Nantes, Nantes, France; 5 UMR 1246 – SPHERE “Methods in Patient-centered outcomes and health research”, Université Nantes et Tours, Nantes, France

**Keywords:** Equimolar mixture of oxygen and nitrous oxide (EMONO), Substance-related disorders, Observational study

## Abstract

**Background:**

Many studies were conducted to assess the benefit/risk ratio of EMONO (Equimolar Mixture of Oxygen and Nitrous Oxide) in France before it was authorized for use outside the hospital setting in 2009. The main objective of this project is to evaluate the effects sought and felt by children when EMONO is used in paediatric dentistry. The secondary objectives are to (i) evaluate the appreciation of EMONO by the children, (ii) characterize children who experience both analgesia and anxiolysis, (iii) evaluate children’s appetite for EMONO and characterize children with a high appetite and (iv) evaluate the impact of the difference in practice among the French dental service university hospitals on anxiety. The maintenance of a framework for the safe use of this drug, whose place in dental care is fundamental, is essential. Twelve of the 16 French dental service university hospitals agreed to participate in this study.

**Methods:**

MEOPAeDent is an observational, descriptive, transversal study that aims to evaluate the effects sought and felt by children when EMONO is used in paediatric dentistry. Subjects requiring dental care under EMONO are recruited by 12 French dental service university hospitals. Patients aged from 3 to 15 years are recruited for the study when they visit a dental service of a French university hospital requiring dental care under EMONO. The investigator collects the necessary data from the child’s medical records, from his own observations and from questions posed to the child and his/her parents. A survey is completed at the first and final sessions of dental care under EMONO.

**Discussion:**

This study will provide an evaluation of the effects of EMONO on the French paediatric population in need of dental care as well as evaluate the appetite for the use of this substance. The results will first be used to provide additional data that is essential to monitor the use of a product with an authorization to use it outside of hospitals from 2009 in France, confirm its safety for use and justify its framework of application.

**Trial registration:**

ClinicalTrials.gov ID: NCT03453411 registered 2 March 2018.

## Background

EMONO is an equimolar mixture of oxygen and nitrous oxide available in France for dental care. It has an analgesic effect with a reduction of the perception threshold of different painful stimuli and an anxiolytic effect. It causes modifications in consciousness: patients are relaxed, sedated and less aware of their surroundings. Pulmonary absorption and elimination of nitrous oxide occur very quickly because of its low solubility in blood and tissues, explaining the rapidity of the analgesic effect and quick return to the initial status after discontinuation of inhalation [1–5].

Based on the product characteristics summary available in the French government’s public drug database [base-donnees-publique.medicaments.gouv.fr/], EMONO indications in dentistry are dental care for infants, children and adolescents, as well as for anxious and patients with disabilities. Administered in combination with oxygen, it improves the cooperation of patients during anxiety-related or painful medical and paramedical procedures (mild to moderate pain) and offers a short duration per use. EMONO in dental care provides great benefit to patients with behavioural disorders, mental retardation or excessive anxiety. Patient cooperation tends to improve under EMONO and, when not used in conjunction with psychotropic agents [6–8], the use of EMONO is safe.

In France, ANSM (French National Agency for Medicines and Health Products Safety) has classified EMONO as a drug known to produce dependency. EMONO obtained its approval in 2001 in France in several indications, particularly in dental care, in hospitals exclusively, for children and patients with anxiety or disability. Its use was considered as very safe in this context [9, 10]. In 2009, the authorization to use it outside the hospital setting has led to a risk management plan implementation. The Nantes CEIP-A (Centers for the Evaluation of and Information on Drug Dependence) has been entrusted with EMONO supervision. It was shown that nitrous oxide could cause problematic consumption: 31 cases of problematic consumption of nitrous oxide alone or in a mixture with oxygen were reported over a period from 1978 to 2014 [11–37]. Classical vulnerability factors of addiction have been reported (family context, psychiatric vulnerability). Several problematic consumer groups with different characteristics have been defined: the first group includes users taking pure nitrous oxide for recreational purposes, the second group is characterized as nitrous oxide users taking it for medical purposes, and the third group is characterized as those taking it in the presence of health professionals, mainly dentists using the nitrous oxide/oxygen mixture [11–37]. However, none of the patients in the ANSM reports were described as those with dependence or abuse due to EMONO use in dental care. The desired effects in patients during EMONO use are relative anxiolysis and analgesia with a preserved state of awakening [2].

The dental service of university hospitals often represent one of the last resorts for dental care at the regional level for patients with limited cooperation because of their disabilities or anxiety. The use of EMONO is often implemented several times for each patient. Indeed, very few dentists nearby use EMONO, leading to a large influx of patients at the university hospital service. However, the authorization to use it outside the hospital setting will lead to an increasing number of dentists using EMONO.

EMONO in paediatric dental care is particularly useful in which EMONO can be administered as the first psychoactive substance known for its positive effects (euphoria). Health professionals observe children who take EMONO daily. The main objective of this project is to evaluate the effects sought and felt by children when EMONO is used in paediatric dentistry. The secondary objectives are to (i) evaluate the appreciation of EMONO by a child, (ii) characterize children who experience both analgesia and anxiolysis, (iii) evaluate children’s appetite for EMONO and characterize children with high appetite and (iv) evaluate the impact of various product applications among the French dental service university hospitals on anxiety. The maintenance of the framework for the safe use of this drug, which is placed in dental care as fundamental, and the benefit ratio as very favourable, is essential. Twelve French dental service university hospitals have agreed to set up this study.

## Methods

### Study design

This observational, descriptive, transversal study aims to evaluate the effects sought and felt by children when EMONO is used in paediatric dentistry. Subjects needing dental care under EMONO were recruited by 12 French dental service university hospitals. Thereafter, the data are collected by practitioner observation, direct interview after dental care under EMONO and using the subject’s medical record.

This project received the financial support from the French National Agency for Medicines and Health Products Safety (ANSM) as part of the 2017 ‘national call for tender’ and also received funding from the local Scientific Committee of the Faculty of Dental Surgery in Nantes as part of the 2017 call for tender.

### Setting of the study

The Dental Service of Nantes University Hospital, along with the Nantes CEIP-A, is the coordinating investigation centre in charge of the general management, monitoring and coordinating of the project. A consortium was set up with partner dental service university hospitals in charge of coordinating the project at the local level in their research area (dental service university hospitals in Brest, Bordeaux, Toulouse, Lyon, Paris Descartes, Nancy, Lille, Reims, Nice, Marseille and Strasbourg). The dental service university hospitals participating in the study are spread over the metropolitan territories of France. The period of inclusion for each participating dental service university hospital is 12 months, and there is no follow up after the patient’s assessment and his/her final care session.

For this national multicentric study, a multi-disciplinary steering committee, comprising pharmacologists, dentists, psychiatrists, methodologists, biostatisticians and clinical research staff, was set up to draft the study protocol. Its mission is to ensure the study’s validity in terms of the scientific content and methodology. The leading committee will handle any practical problems occurring during the study and where appropriate, decide on amendments to the protocol. The committee shall meet regularly, and whenever necessary, to approve aspects concerning practical organization and to answer any questions raised during the study.

### Participants

#### Eligibility criteria

The patients are recruited for the study by their dentist when they visit the dental service university hospitals for their dental care under EMONO, according to the following inclusion criteria:

- Children aged 3 to 15 years
- Requiring dental care under EMONO
- Being able to provide the details to answer the questionnaire of the study
- Cooperating along with his/her parents and ready to participate in the study

The exclusion criteria of the study are as follows:

Patients are restricted from participation if their dental care can be performed in a vigilant state or involves several important treatments, justifying general anaesthesia. Finally, children who cannot understand the questionnaire will not be allowed to participate.

### Patient recruitment

Each dentist from a dental service university hospital participating in the study recruits patients needing dental care under EMONO over a 12-month period. If the patient meets all the inclusion criteria, the dentist asks the child and his or her parents or legal guardian, if he or she wishes to participate in the study, providing clear and appropriate verbal and written information on the study (objectives, scientific interest, and practical procedures).

Without any delay, the dentist collects the patient’s oral consent (as well as that from at least one of the parents or legal guardians). The lack of delay is due to the design of the study, which has been approved by the Committee for the Protection of Persons (CPP).

### Consent for participation

At inclusion, the patients receive an information leaflet for their age range, as well as their parents or legal guardian. The investigator will note in the patient’s file that the patient and parents have been orally informed, received the briefing note and gave their oral consent to participate in the research. At least one parent of all children, regardless of the age of the child, must be informed and must consent.

### Ethics statement

This non-interventional study has been declared to the French Data Protection Body (CNIL) and received a favourable opinion from the Committee for the Protection of Persons. It has also been registered at ClinicalTrials.gov.

### Variables and data measurement

Data for the analysis are collected in two steps, through the survey completed by the dentist at both the first and final sessions of dental care under EMONO. If the patient requires only one treatment session, only the first part of the questionnaire is completed.

### Survey

Once the investigator receives oral consent to participate in the research from the patients and at least one of the parents (or legal guardian), the necessary data are collected from the child’s medical records, from his own observations and from questions to the child and his/her parents. A survey is completed at both the first and final sessions of dental care under EMONO.

#### Primary endpoint

Our primary endpoint, to describe the effects of EMONO that are felt and sought during care during the first and last treatment sessions, will be performed by questioning the patients comprehensively about the presence or absence of each expected effect of EMONO (waking state preserved, no abolition of judgment, relative analgesia, anxiolysis), and each adverse event (slight decrease in consciousness, agitation, anxiety, euphoric effect, nausea or vomiting, sensation distortion) at the first and last treatment sessions. The presence of unexpected “other” effects will also be indicated. When scored as present, all effects will be considered as experienced by the patient.

For each effect felt, the patient will be asked about his or her perception of the effect (positive or not).

To describe the effects felt and sought with EMONO use, the prevalence of each effect felt will be calculated, and the proportion of patients who felt this effect and found it pleasant (positive) will also be indicated.

#### Secondary endpoints

*Three parameters will be used to evaluate the child’s assessment of EMONO during the first session of care:*

- VAS (Visual analogue scale): the patient marks on the line the point he/she feel represents their perception and eventually their appreciation of contact with EMONO
- Choice of the child with an adapted drug-liking test: the patient will be asked if he/she prefers to exchange the toy that he/she traditionally receives at the end of the care against an EMONO inhalation without care [38, 39]
- The child’s will to continue care in the case of adverse effects

*Three parameters will define children who experience both analgesia and anxiolysis during the first session of care:*

- Decreased score on the anxiety scale (Venham modified by Veerkamp) before and after inhalation of EMONO
- Presence of anxiolysis among the effects felt
- Presence of analgesia among the effects felt

We will estimate the prevalence of children for whom at least one of the first two parameters and the third parameter are present during the same session of EMONO use.

*The parameter “Presence of signs that the child is trying to prolong contact with EMONO” will be used to define patients with a higher appetite for EMONO (shown by patients’ attempt to extend the duration of EMONO use physically or verbally) during the first session of care. The following three parameters will characterize these children:*

- Table answering the questions about the presence of expected effects of EMONO and their perceptions
- Medical history
- Family situation of parents and anxiety of parents [40–42]

*Three parameters will make it possible to evaluate the impact of the difference of practice between the French dental service university hospitals regarding the anxiety during the first and last sessions of care:*

- Decreased score on the anxiety scale (Venham modified by Veerkamp) before and after inhalation of EMONO
- Use of a sedative premedication
- Patient management using closed boxes or open spaces

The different practices evaluated in the French dental service university hospital will include the use or absence of a sedative premedication and management of the patient in closed or open boxes.

The evolution of the EMONO intake between the first and last sessions of care will be evaluated by the existence of an amnesic effect during the last session, tolerance during the history of care (noticed during the last session), difference in the effective dosage between the first and last sessions and the regular concern of the child for EMONO consumption according to the parents during the last session. The difference in EMONO appetite between the first and last sessions will also be recorded.

### Study size

The evaluation of the effects sought and felt by children when EMONO is used in paediatric dentistry does not require any sample size calculation. The dental service of Nantes University Hospital manages between 8 and 10 patients eligible for research per week and has a national average of numbers supported by participating dental services.

This estimate allows a 12-month inclusion potential, which represents between 5000 and 6000 patients. Considering a ratio of one eligible patient out of three to four to include, the recruitment aim is 1300 patients. The period of inclusion was set at 12 months to ensure that the study would be proposed to a maximum of eligible patients.

### Statistical methods

All the evaluation parameters will be subjected to a descriptive analysis. The quantitative variable evaluation parameters will be described using position parameters (mean or median) and dispersion (standard deviation, interquartile range and range). Normality of their distribution will be assessed (normality test and evaluation using the relevant graphic tools). The qualitative variable evaluation parameters will be shown in the form of number and frequency tables for each modality.

To answer the main objective (description of the effects felt and sought using EMONO), the prevalence of each effect felt will be calculated, and the proportion of patients who felt this effect and found it pleasant (positive) will also be indicated.

Each criterion to evaluate the EMONO’s assessment by the child will be analysed independently (average for the VAS score, prevalence of drug-liking and continuation of care).

The prevalence of children who experience both analgesia and anxiolysis will be estimated. A multivariate logistic regression model will be applied to define the associated factors: child/parent socio-demographic characteristics and antecedents.

The prevalence of children with a higher appetite for EMONO will be estimated. A multivariate logistic regression model will be applied to define the associated factors: socio-demographic child/parent characteristics, medical history, presence of expected EMONO effects and their perceptions.

The impact of care practices (sedative premedication and closed/open box management) on anxiety will be evaluated using average comparisons (Student’s tests) of the anxiety score before and after the inhalation of EMONO. A comparison will also be made on the difference in the pre- and post-inhalation score.

The evolution of EMONO intake between the first and last sessions will be described in terms of the prevalence (amnesic effect, tolerance, concern), appetite difference and difference between the effective dosage at the first and last treatment sessions will be calculated.

## Discussion

A pilot study was carried out at the Dental Service of Nantes University Hospital, considering most of the parameters that will be used for this national study. It revealed a relatively small number of patients with both anxiolysis and analgesia effects. Sixty-two percent of patients presented an anxiolytic effect, and 40% showed relative analgesia. Both effects were associated in 33% of children. These effects were among the most monitored in patients [43].

Among the 76 patients assessed, 12 attempted to extend the duration of EMONO use (16%), which was considered similar to signalling a higher appetite for the product. After bivariate statistical analysis, none of the variables collected appeared significantly associated with the extension of EMONO use.

Recruiting patients needing EMONO for dental care will not be a challenge for the partners working on the project. The main objective of this project is to evaluate the effects sought and felt by children when EMONO is used in paediatric dentistry. It does not need a precise number of subjects. On the other hand, being able to characterize patients with a higher appetite for EMONO will require 1291 patients from a subject calculation with an alpha risk of 5%. This calculation was made from the results of the pilot study.

The purpose of the study is to evaluate the use of EMONO received only at the hospital. In addition to considering elements of the subject’s medical and family histories, this evaluation includes a semi-structured interview with the subject after its consumption. Given the variability in the study population, the interview will be adapted to the child’s or adolescent’s age, maturity and comprehension skills. The subject’s parent or legal guardian may also be questioned in addition to the subject.

In paediatric dentistry, the use of EMONO in children corresponds in most cases to the first administration of a substance known for its positive effect, euphoria. It seems fundamental to us to evaluate the therapeutic and non-therapeutic effects to better estimate the benefit/risk ratio of the administration. The interest of this evaluation is such that most of the French dental service university hospitals (12/16) responded favourably and wishes to participate in this work. A national study would increase the power and ensure the representativeness of the data collection.

MEOPAeDent is the first study that proposes to evaluate the appetite for the use of EMONO in the French paediatric population in need of dental care, in addition to evaluate the effects sought and felt by children. We believe that this substance evaluation known for its very important contribution in paediatric dentistry will confirm its safety of use and justify its framework of application.

## Abbreviations

ANSM: French National Agency for Medicines and Health Products Safety
CEIP-A: Centers for the Evaluation of and Information on drug Dependence
CNIL: French Data Protection Body
EMONO: Equimolar Mixture of Oxygen and Nitrous Oxide
VAS: Visual analogue scale
